# P-1139. Surveying Antibiotic Treatment for Pediatric Patients with Suspected Urinary Tract Infections at Madigan Army Medical Center

**DOI:** 10.1093/ofid/ofae631.1326

**Published:** 2025-01-29

**Authors:** Samantha Lonergan, Rebecca Sainato, Blake Cirks

**Affiliations:** Madigan Army Medical Center, Tacoma, Washington; Madigan Army Medical Center, Tacoma, Washington; Madigan Army Medical Center, Tacoma, Washington

## Abstract

**Background:**

Urinary tract infections (UTIs) are one of the most common bacterial infections in pediatric patients presenting to the Emergency Department (ED). However, the gold standard for diagnosis is a urine culture which takes 24-48 hours to result leading to antibiotics started empirically while results are pending. This study was implemented with the primary aim of evaluating the antibiotic management of pediatric patients who were seen at a single medical center ED with suspected UTI.

**Figure:**
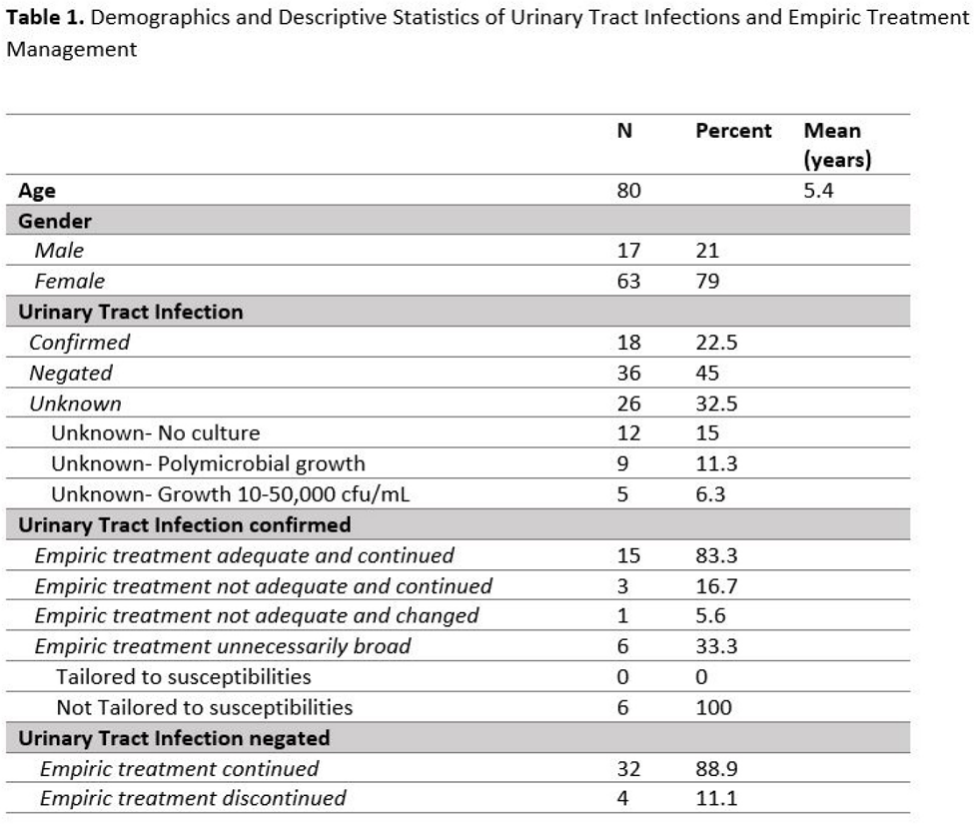

**Methods:**

Using the electronic medical record we identified pediatric cases (ages 2 months-18 years) where UTI was diagnosed in the ED during 2023 using the ICD-10 code. Data collected on each case included demographics, laboratory work-up, prescribed medications, and follow-up. Outcomes were categorized as confirmed, unknown, or negated. A confirmed UTI required an abnormal urinalysis and single species growth exceeding 50,000 cfu/mL; UTI was negated when growth was < 10,000 cfu/mL. Descriptive statistics and comparison tests were used as appropriate.

**Figure:**
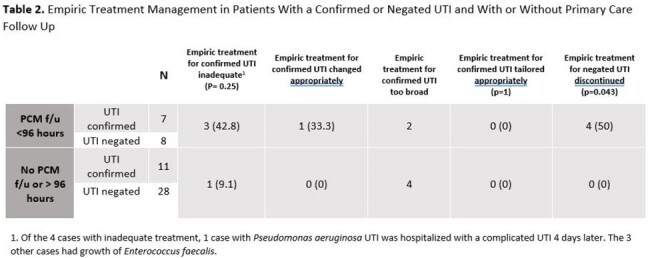

**Results:**

There were 80 UTI diagnoses in 70 patients. 18 (22.5%) cases were confirmed and 36 (45%) were negated (Table 1). We analyzed 54 cases with confirmed or negated UTIs: 15 (27.8%) had follow-up with primary care (PCM) within 96 hours of their ED visit. When UTI was negated, 4 cases (11%) had antibiotics discontinued and all had PCM follow-up within 96 hours (p=0.043) (Table 2). *Enterococcus*-UTI accounted for 75% of cases where empiric treatment was inadequate. Of those without confirmed UTI, 3 returned to the ED with adverse effects from antibiotics and 2 with multiple inconclusive evaluations were referred to urology. The ED providers notified families with positive urine culture results, but no one was notified if the urine culture was negative.

**Conclusion:**

Many pediatric patients who are treated for UTI in our ED have inconclusive or negative workups resulting in unnecessary antibiotic use, ED visits for antibiotic complications, and subspecialty referrals. Our current system does not support the discontinuation or tailoring of unnecessary or inappropriate antibiotics unless patients have close follow-up. Antibiotic stewardship requires systematic support within the ED to limit unnecessary antibiotics and the inherent complications that follow.

**Disclosures:**

**All Authors**: No reported disclosures

